# Ocular toxicities associated with anticancer drug therapy in Asian patients: a literature review and practical management recommendations for non-ophthalmologic settings

**DOI:** 10.1007/s10147-026-03096-x

**Published:** 2026-06-18

**Authors:** Yohei Iimura, Hitomi Sakai, Isana Nakajima, Seiichiro Kuroda, Michio Nakamura

**Affiliations:** 1 Department of Pharmacy, The IMSUT Hospital, The Institute of Medical Science, The University of Tokyo, 4-6-1, Shirokanedai, Minato-ku, Tokyo 108-8639 Japan; 2 Advanced Cancer Translational Research Institute, Showa Medical University, Tokyo, Japan; 3 Department of Ophthalmology and Visual Science, Kochi Medical School, Kochi University, Kochi, Japan; 4 Department of Gastroenterology, Sapporo City General Hospital, Sapporo, Hokkaido Japan

**Keywords:** Anticancer therapy, Ocular toxicity, Molecular targeted therapy, Immune checkpoint inhibitors, Antibody–drug conjugates, Asian population

## Abstract

**Background:**

Ocular toxicities associated with anticancer therapies, including cytotoxic chemotherapy, hormonal agents, molecular targeted therapies, immune checkpoint inhibitors, and antibody–drug conjugates, are increasingly recognized and may substantially impair visual function and quality of life. However, ethnicity-specific data for Asian populations and practical management strategies applicable to non-ophthalmologic settings remain limited.

**Methods:**

A literature review of publications up to December 2025 was conducted using PubMed to identify studies reporting incidence, mechanisms, prevention, or management of anticancer drug–associated ocular toxicities in Asian patients. Based on the extracted evidence, practical management recommendations applicable to healthcare settings without on-site ophthalmologists were developed.

**Results:**

Overall, 155,202 manuscripts were identified, of which 70 studies met the inclusion criteria. Most of these manuscripts were case reports, retrospective analyses, or expert recommendations, with few prospective or interventional studies. Ocular toxicities varied by anticancer drug class and involved the ocular surface, retina, optic nerve, and immune-mediated inflammation. Incidence and clinical characteristics differed across drug classes, and ethnicity-specific data for Asian patients were inconsistently reported. Based on these findings, a symptom-based management algorithm applicable to non-ophthalmologic settings was developed, emphasizing early recognition of ocular toxicities, supportive care (e.g., artificial tears and topical steroids) initiation, treatment interruption for persistent or moderate symptoms, and timely ophthalmology referral for visual acuity decline or refractory cases.

**Conclusions:**

This review summarizes current evidence on anticancer drug–associated ocular toxicities in Asian patients and proposes pragmatic recommendations and an algorithm to support their early recognition and management in non-ophthalmologic settings.

## Introduction

Cancer drug therapy has markedly evolved over recent decades, expanding from classical cytotoxic agents (CTx) and hormonal therapies to molecular targeted agents (MTAs), immune checkpoint inhibitors (ICIs), and antibody–drug conjugates (ADCs). However, traditional CTx and hormonal therapies have been associated with various ocular toxicities that may adversely affect visual function and quality of life (QOL). For example, tamoxifen has been linked to keratopathy, retinopathy, and optic neuritis [1, 2]; taxane-based regimens such as paclitaxel and docetaxel have been associated with increased risks of epiphora, cystoid macular edema, and optic neuropathy [3]; and systemic use of 5-fluorouracil and other cytotoxic combinations causes conjunctival and corneal irritation, tearing, and blurred vision. The advent of targeted agents and immunotherapies has introduced new challenges. MTAs and ICIs have been associated with a broad spectrum of ocular toxicities, affecting the eyelid, conjunctiva, cornea, retina, optic nerve, and uvea. These events may range from dry eye and uveitis to more severe immune-related ocular conditions, requiring prompt recognition and management [4]. ADCs, a rapidly growing cancer therapeutic class designed to deliver cytotoxic payloads directly to tumor cells, have also been linked to significant ocular toxicities. A systematic review and meta-analysis of ADC clinical trials reported a pooled incidence of ocular toxicities of approximately 10.4%, with dry eye being the most common manifestation [5]. Furthermore, real-world pharmacovigilance data highlight that ADC-related corneal disorders and other ocular toxicities are frequently reported and may develop relatively early during therapy, underscoring their clinical importance [6]. These ocular toxicities, although often non-life-threatening, can substantially impair daily activities, reduce adherence to cancer therapy, and diminish patients’ QOL. Symptoms such as blurred vision, eye pain, photophobia, and visual field disturbances can lead to dose delays, reductions, or discontinuations, potentially compromising anticancer efficacy. Therefore, understanding the full spectrum of ocular toxicities across different anticancer agent classes is essential for achieving comprehensive patient care.

An additional consideration in ocular toxicity research is the potential influence of ethnic and anatomical differences. Ethnic variations in pharmacokinetics, potentially driven by genetic polymorphisms, body composition, and environmental factors, may contribute to interpopulation variability in drug response and safety profiles. Specific Asian populations have been reported to exhibit anatomical variations, such as thicker soft tissue in the upper eyelids and a higher prevalence of epicanthal folds. Furthermore, studies suggest that Asian eyes may have a shallower anterior chamber, narrower drainage angles, and potentially thinner central corneal thickness, compared with other ethnic groups. While these characteristics are often discussed in clinical settings, their direct impact on anticancer drug-induced ocular toxicities remains unclear and should be interpreted as hypothesis-generating. Additionally, studies indicate that Asian eyes have longer axial length [7, 8]. Moreover, the prevalence of myopia, which affects ocular structure and visual function, is particularly high in many East Asian populations, further contributing to differences in ocular physiology and disease risk [9]. Vogt–Koyanagi–Harada (VKH)-like uveitis has been strongly associated with HLA-DR4, particularly HLA-DRB1*04 alleles, which are more prevalent in Asian populations. This immunogenetic background may partly explain the higher susceptibility to drug-induced immune-mediated uveitis in Asian individuals [10–12]. These anatomical and physiological differences suggest that the frequency, presentation, and optimal management of cancer therapy-related ocular toxicities may differ between Asian and non-Asian populations.

Moreover, the incidence and severity of ocular toxicities can be influenced by ethnic differences due to drug metabolism. Fluoropyrimidine-based agents, particularly S-1, are more widely used in clinical practice in Asian countries than in Western countries. This is partly due to the genetic polymorphism of CYP2A6, a major enzyme responsible for converting tegafur to 5-FU. Asian populations have a higher prevalence of low-metabolizer alleles (e.g., CYP2A6*4), which cause slower conversion and different pharmacokinetics compared with those of Western populations [13, 14]. In Western patients, severe gastrointestinal toxicities often act as a dose-limiting factor, leading to earlier treatment discontinuation or dose reduction. Conversely, Asian patients tend to better tolerate these systemic effects, allowing for longer treatment durations and higher cumulative drug exposure, thereby increasing the likelihood of developing cumulative adverse events, such as lacrimal duct obstruction and corneal epithelium disorders, making proactive ocular management a critical priority in Asian oncology settings. However, data specifically focusing on ocular toxicities in Asian patients receiving cancer drug therapy remain limited.

Recently, several large-scale analyses and comprehensive reviews have addressed the incidence and management of MTA-associated ocular toxicities, providing valuable insights into drug-specific risk profiles and management strategies, including recommendations for detailed ophthalmologic examinations and specialized diagnostic testing [15–20]. While these reports have substantially advanced the understanding of MTA-related ocular toxicities, many of the proposed management strategies rely on advanced ophthalmologic assessments, such as slit-lamp biomicroscopy, optical coherence tomography, and fundus examinations, which may not be readily available in routine oncology practice. Therefore, the applicability of these recommendations is limited in settings where ophthalmologists are not routinely available or access to specialized ophthalmologic equipment is restricted. Such scenarios are common in community hospitals and regional cancer centers, particularly in regions of Asia, where oncologists are often required to manage ocular symptoms based primarily on patient-reported complaints and basic clinical examination findings. Consequently, there remains a substantial gap between published management recommendations and real-world clinical practice for cancer therapy–related ocular toxicities.

Furthermore, although management strategies for MTA-associated ocular toxicities have been relatively well described, no comprehensive management recommendations have been systematically established for ocular toxicities induced by classical CTx. This gap is notable given that CTx continue to be widely used and are frequently administered in combination with newer therapies, potentially compounding ocular toxicity risks. The lack of practical, evidence-based guidance for managing ocular toxicities related to CTx further complicates clinical decision-making, particularly in non-ophthalmologic settings.

Therefore, in addition to clarifying the incidence and spectrum of cancer drug–related ocular toxicities in Asian populations, this literature review aims to bridge this critical gap by synthesizing available evidence to propose practical and accessible management recommendations. The review focuses on strategies that can be implemented in medical institutions without on-site ophthalmologists or advanced ophthalmologic diagnostic capabilities, thereby supporting safer and more consistent management of ocular toxicities in real-world oncology practice.

Furthermore, the rationale for focusing on Asian populations extends beyond biological and pharmacogenomic diversity. Across Asian countries, there are substantial variations in healthcare systems, insurance coverages, and specific oncological prescribing practices. Moreover, access to routine or urgent ophthalmologic evaluations varies significantly between urban and rural areas or among different regions in Asia. These healthcare systemic factors, combined with distinct patterns of anticancer drug utilization, strongly underscore the clinical importance of establishing practical, accessible ocular toxicity management recommendations tailored to non-ophthalmologic settings in Asia.

## Patients and methods

### Literature search and selection

A literature review was conducted to identify publications reporting anticancer drug–associated ocular toxicities, focusing on incidence, clinical characteristics, management strategies, prevention, and treatment. Specifically, PubMed was searched for relevant articles published up to December 2025. The search strategy was designed to comprehensively capture reports related to cancer therapy–associated ocular toxicities and their management, and the following keywords and their combinations were used: “anticancer therapy,” “chemotherapy,” “molecular targeted therapy,” “immune checkpoint inhibitor,” “antibody–drug conjugate,” “ocular toxicity,” “ocular adverse events,” “ophthalmic toxicity,” “eye disorders,” “visual impairment,” “visual field defect,” “incidence,” “frequency,” “management,” “treatment,” and “prevention.” These terms were combined using Boolean operators (AND/OR) as appropriate.

Eligible articles included original research, retrospective and prospective observational studies, clinical trials, case series, case reports, and narrative or systematic reviews that described anticancer drug–related ocular toxicities. Reports on incidence, clinical presentation, management strategies, preventive measures, or treatment outcomes were considered relevant. Articles were excluded if they were unrelated to anticancer drug therapy, did not describe ocular toxicities, or lacked sufficient clinical information relevant to incidence or management. Non-English–language articles were excluded unless they contained an English abstract providing sufficient detail for data extraction. Conference abstracts without full-text availability were also excluded. Titles and abstracts were initially screened to assess relevance, followed by a full-text review of potentially eligible articles. From each included publication, data were extracted regarding the anticancer agent type, reported ocular toxicities, incidence or frequency (when available), management or preventive strategies, and clinical outcomes. Particular attention was paid to studies involving Asian populations, as well as to those addressing management approaches applicable in settings without access to specialized ophthalmologic evaluation.

In this review, the term ‘Asian population’ primarily encompasses individuals of East, South, and Southeast Asian ethnicity. For the purpose of data extraction, studies conducted within Asia or those providing specific subgroup analyses of Asian participants were included. For global trials with mixed populations, data were preferentially extracted from reported Asian subgroup analyses to ensure the relevance of our findings to the target population.

## Results

### Text description

**Fig. 1:**
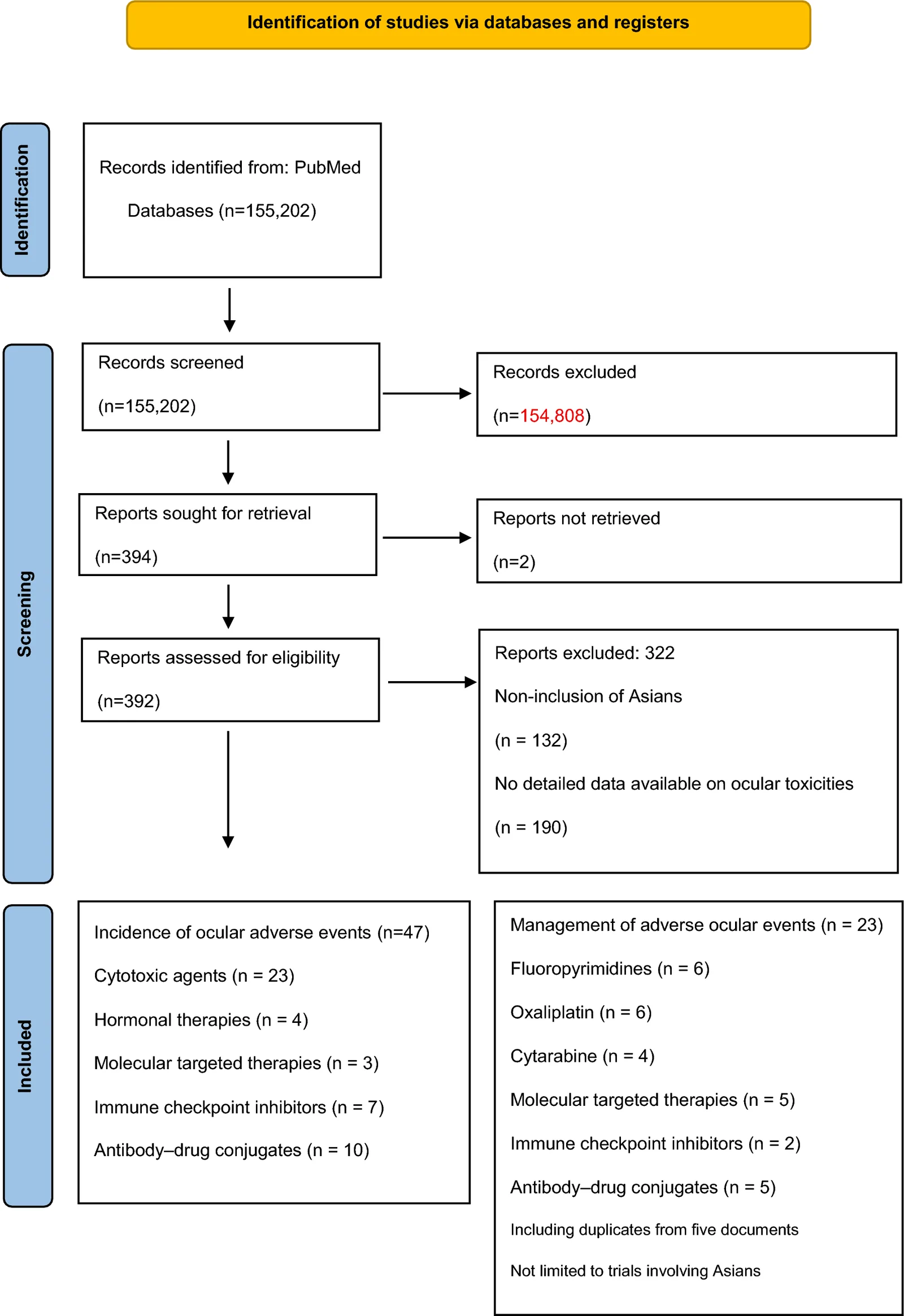
Diagram of the literature selection/screening process

The PubMed search identified 155,202 records published up to December 2025. After removal of duplicates, 392 remained for title and abstract screening. Of these, 132 records were clinical studies that did not include Asians, and 190 articles were excluded because they did not report the incidence or management of ocular toxicities. Ultimately, 70 studies were included in the literature review (Fig. 1).

Our literature review identified a wide spectrum of ocular toxicities affecting various anatomical structures of the eye. For the benefit of non-ophthalmological clinicians, we have summarized these toxicities based on their clinical presentations. Table 1 provides a comprehensive classification of anticancer drugs categorized by common ocular symptoms, such as excessive tearing, blurred vision, and eye redness. This classification aims to assist healthcare providers in non-ophthalmologic settings for identifying potential drug-induced causes during routine patient consultations. Terminology used in this review follows the Common Terminology Criteria for Adverse Events (CTCAE) version 5.0 to ensure consistency with standard oncological practice. Toxicities are further classified into the anterior segment (e.g., the cornea and conjunctiva), posterior segment (e.g., the retina), and ocular adnexa (e.g., lacrimal system and the eyelids) to clarify the anatomical focus of each drug’s effect.

**Table 1:** Summary of anticancer drugs categorized by ocular toxicities

Common symptoms	Potential ocular toxicities	Representative drugs
Excessive tearing (Epiphora)	Lacrimal duct stenosis/obstruction	S-1, Capecitabine, Docetaxel, Paclitaxel
Dryness, Grittiness, Redness	Corneal epithelium disorders/Keratitis/Conjunctivitis	S-1, EGFR inhibitors (Gefitinib, etc.), ADCs (Belantamab mafodotin)
Blurred vision/Vision loss	Macular edema/Retinopathy/Cataract/Uveitis	Taxanes, MEK inhibitors, FGFR inhibitors, ICIs, Tamoxifen
Eye pain, Photophobia	Uveitis/Episcleritis	ICIs (Nivolumab, Pembrolizumab, etc.), BRAF inhibitors
Eyelid/Eyelash changes	Trichomegaly/Blepharitis	EGFR inhibitors, Panitumumab, Cetuximab

*S-1* combination of tegafur, gimeracil, *EGFR* epidermal growth factor receptor, *ADC* antibody–drug conjugates, *MEK* MEK(Mitogen-activated protein kinase kinase, *FGFR* fibroblast growth factor receptor, *ICI* immune checkpoint inhibitor, *BRAF* v-raf murine sarcoma viral oncogene homolog B1

### Fluoropyrimidines

Ocular toxicities were most frequently reported with S-1 and capecitabine. In S-1–treated patients, lacrimal drainage obstruction and corneal epithelial injury were described, primarily in studies conducted in Japan. Although no prospective trials quantified the incidence, experimental and translational studies demonstrated an association between tear tegafur concentration and ocular toxicities [21]. In an animal model, repeated artificial tear administration reduced S-1–induced corneal surface injury [22]. Regarding capecitabine, ocular toxicities were rare and limited to isolated case reports. The reported manifestations included visual impairment [23], bilateral ectropion [24], conjunctivitis [25], and corneal toxicity [26, 27].

### Platinum agents

Among platinum compounds, oxaliplatin was the most frequently implicated agent. A retrospective study reported ocular toxicities in fewer than 5% of patients treated with oxaliplatin-based chemotherapy [28]. Additionally, multiple case reports described transient or persistent visual loss and visual field defects temporally associated with oxaliplatin [29–33]. However, these reports did not provide pooled incidence rates but consistently described symptom onset during or shortly after treatment.

### Antimetabolites

Cytarabine-associated ocular toxicities, particularly conjunctivitis and keratitis, were consistently reported in clinical studies. Several reports showed that the incidence and severity of ocular symptoms were markedly reduced with the use of prophylactic or therapeutic steroid eye drops [34–37]. Although ethnicity-specific incidence data were not provided, ocular toxicity was described as a common adverse event in the absence of prophylaxis.

### Taxanes and hormonal agents

Regarding taxanes (paclitaxel and docetaxel), epiphora, cystoid macular edema, and optic neuropathy were reported. Epiphora was reported in up to 10–20% of patients in some cohorts, although these data were derived mainly from Western populations [3]. For the hormonal agent tamoxifen, ocular toxicities such as keratopathy, retinopathy, and optic neuropathy were reported in relation to cumulative dose and treatment duration. These events were described as uncommon at standard doses, and ethnicity-stratified incidence data were unavailable [1, 2].

### ICIs

ICI-associated ocular immune-related adverse events have been infrequently reported, and documented manifestations include uveitis, conjunctivitis, and ophthalmoplegia. The overall incidence has been generally reported to be less than 1–2% across studies, although variability exists depending on cancer type and treatment. Notably, in patients receiving immune-based therapies for malignant melanoma, uveitis develops relatively frequently and is thought to result from T-cell–mediated cross-reactivity against melanocyte-associated antigens shared by melanoma cells and uveal tissues [38, 39]. Specifically, a higher ophthalmoplegia frequency was reported in Asian lung cancer cohorts [40], and responses to topical corticosteroids were described in case-based reports [41, 42].

### MTAs

Regarding MTAs, including epidermal growth factor receptor (EGFR) and vascular endothelial growth factor (VEGF) inhibitors, ocular surface disorders such as dry eye, keratitis, and conjunctivitis have been commonly reported. However, the incidence varies widely across agents and studies, and precise quantitative data are not consistently available. Furthermore, BRAF/MEK inhibitors have been associated with MEK inhibitor–associated retinopathy, characterized by reversible serous retinal detachment–like changes [46–48]. In contrast, drug-induced uveitis, which is mediated by inflammatory mechanisms, has also been reported independently of MEK inhibitor–associated retinopathy [10–12]. Ethnicity-specific incidence data were not systematically reported [43].

### ADCs

Among all drug classes, ADCs were the only agents for which ocular toxicity incidence was clearly quantified in phase III trials. In the FORWARD I and MIRASOL phase III trials of mirvetuximab soravtansine, ocular toxicities were reported in a substantial proportion of patients. Approximately 42% of patients developed blurred vision, and keratopathy developed in approximately 32.5% (any grade), with grade ≥ 3 events occurring in a minority of patients. Overall ocular toxicity was reported in approximately 56% of patients in the MIRASOL trial, with grade 3 events occurring in approximately 14% [44–47]. Reported incidence varies substantially depending on study design. For several agents, particularly molecular targeted therapies and antibody–drug conjugates, incidence data are derived primarily from observational studies, post-marketing reports, or expert consensus rather than prospective trials. Ethnicity-specific incidence data remain limited (Table 2).

**Table 2:** Reported ocular toxicities associated with anticancer agents: evidence from observational studies and case reports

References	Drug class	Drug	Reported ocular toxicities	Reported incidence/frequency	Population notes (Asian vs. others)
[21] Cohort study	Fluoropyrimidines	S-1	Lacrimal drainage obstruction, corneal epithelial injury	Frequently occurred in Japanese patients	High proportion of reports from Japan; 40.4% of patients developed S-1 related ocular toxicities
[23–27] Case report		Capecitabine	Visual impairment, conjunctivitis, corneal toxicity, ectropion	Rare; limited to isolated case reports	Cases reported from both Asian and non-Asian populations
[28–33] Retrospective study and case report	Platinum agents	Oxaliplatin	Transient or persistent visual loss, visual field defects	≤ 5% in retrospective analyses; mostly transient	Retrospective data mainly from Asian cohorts; similar patterns in Western case reports
[34–37] Retrospective study and case report	Antimetabolites	Cytarabine	Conjunctivitis, keratitis	Common without prophylaxis; markedly reduced with steroid eye drops	No clear ethnicity-specific difference reported
[3] Retrospective study	Taxanes	Paclitaxel/Docetaxel	Epiphora, cystoid macular edema, optic neuropathy	Epiphora reported in 10–20% of patients in observational cohorts	Large cohort data mainly from Western populations
[1, 2] Prospective observational study	Hormonal agents	Tamoxifen	Keratopathy, retinopathy, optic neuropathy	Dose- and duration-dependent; uncommon at standard doses	No ethnicity-stratified incidence reported
[40–42] Case report	Immune checkpoint inhibitors	Anti-PD-1/Anti-CTLA-4	Uveitis, conjunctivitis, ophthalmoplegia	Rare (< 1–2%); variable across studies	High frequency (40%) of ophthalmoplegia reported in Asian lung cancer cohorts VKH-like uveitis is more prevalent in Asian populations
[43] Review	Molecular targeted agents	EGFR inhibitors, VEGF inhibitors	Dry eye, keratitis, conjunctivitis, abnormal eyelash Growth, eye pain	Common but usually mild; precise incidence varies by agent	Ethnicity-specific incidence not well quantified
[44–47] Review	Antibody–drug conjugates	Enfortumab vedotin, others	Ocular surface disorders, keratopathy	Frequency not systematically reported; increasingly recognized	Data largely from global trials; Asian-specific data lacking

*S-1* combination of tegafur, gimeracil, and oteracil potassium, *CTLA-4* Cytotoxic T-lymphocyte associated protein 4, *EGFR* epidermal growth factor receptor, *VEGF* vascular endothelial growth factor

### Ocular and visual toxicities in phase III trials of anticancer agents

#### Overview of included trials

Phase III randomized clinical trials reporting ocular or visual toxicities associated with anticancer agents were identified across CTx, hormonal therapy, MTA, ICIs, and ADCs. Only trials in which ocular or visual adverse events were explicitly reported, categorized, or predefined as adverse events of special interest were included in this analysis (Table 3).

**Table 3:** Reported incidence of ocular and visual toxicities in phase III clinical trials of anticancer agents

Drug/Regimen	Study design	Cancer type	Population (Asian %)	Reported ocular/Visual toxicities	Incidence	References
S-1 (adjuvant)	Phase III, randomized	Stage III colorectal cancer	Japanese (~ 100%)	Keratitis; epiphora	Keratitis 5% (grade ≥ 3: 1%); epiphora 19% (grade ≥ 3: 2%)	[48]
Capecitabine (adjuvant)	Phase III, randomized	Stage III colorectal cancer	Japanese (~ 100%)	Keratitis; epiphora	Keratitis 2%; epiphora 5%	[48]
Capecitabine ± bevacizumab	Phase III, randomized	Stage II–III colorectal cancer	Predominantly non-Asian	Blurred vision; dry eye; impaired vision	Blurred vision, 1–2%; Dry eyes, 9–13%	[64]
Oxaliplatin-based (CAPOX/mFOLFOX6)	Phase III, randomized (ACHIEVE)	Stage III colon cancer	Japanese (~ 100%)	Eye disorders (AE category)	Frequency not specified	[49]
Low-dose cytarabine+etoposide ± ATRA	Phase III, randomized	AML	Predominantly European	Ocular/visual (CTCAE category)	ATRA arm: 2 events; control: 0	[65]
Paclitaxel (maintenance)	Phase III, randomized	Ovarian cancer	Asian 8–9%	Ocular/visual	Any grade, 5–6%; grade ≥ 3, < 1%	[66]
Tamoxifen versus toremifene	Randomized ophthalmologic follow-up	Early breast cancer	Predominantly non-Asian	Cataract; macular crystals	Cataract during follow-up 23.8%	[50]
Tamoxifen+fenretinide versus placebo	Phase III, randomized	Breast cancer	Predominantly non-Asian	Visual problems	27% (tamoxifen + fenretinide arm) versus 11.8%; grade 3: 3.5% (tamoxifen + fenretinide arm) versus 0%	[51]
Nivolumab versus chemotherapy (ATTRACTION-3)	Phase III, randomized	Esophageal SCC (2L)	Asian ~ 96%	Uveitis (immune-related; discontinuation criterion)	Frequency not specified	[52]
Pembrolizumab+perioperative chemotherapy (KEYNOTE-585)	Phase III, randomized	Resectable gastric/GEJ adenocarcinoma	Asian 48%	Uveitis (AE of interest)	< 1% (group-specific counts reported)	[53]
Atezolizumab versus best supportive care (IMpower010 Japan)	Phase III (subgroup analysis)	Resected NSCLC	Japanese 100%	Ocular inflammatory toxicity	1.8% (atezolizumab arm) versus 1.7% (grade ≥ 3: 1.7% in BSC)	[54]
Tremelimumab versus dacarbazine/temozolomide	Phase III, randomized	Advanced melanoma	Asian 0%	Ocular infections, irritations, or inflammation	4% (tremelimumab arm) versus 1% (grade ≥ 3: 0 in both)	[67]
Osimertinib versus gefitinib/erlotinib (FLAURA)	Phase III, randomized	EGFR-mutated advanced NSCLC (1L)	Asian 62%	Eye included as baseline metastatic site category	Frequency not specified	[57]
Dacomitinib versus gefitinib (ARCHER 1050)	Phase III, randomized	EGFR-mutated advanced NSCLC (1L)	Asian ~ 76%	Conjunctivitis	19% (dacomitinib arm) versus 4% (grade ≥ 3: 0 in both)	[55]
Belantamab mafodotin versus pomalidomide–dexamethasone (DREAMM-3)	Phase III, randomized	Relapsed/refractory multiple myeloma	East Asian + Japanese ~ 20%	Blurred vision; visual acuity reduced; keratopathy; ocular toxicities	Ocular toxicities 66% (grade ≥ 3: 29%) (belantamab mafodotin arm)	[58]
Belantamab mafodotin+bortezomib+dexamethasone (DREAMM-7)	Phase III, randomized	Relapsed/refractory multiple myeloma	Asian 12–13%	Ocular events category (blurred vision, dry eye, photophobia)	79% (belantamab mafodotin arm) versus 29% (grade ≥ 3: 34% vs. 3%)	[59]
Belantamab mafodotin+pomalidomide+dexamethasone (DREAMM-8)	Phase III, randomized	Relapsed/refractory multiple myeloma	Asian 12–13%	Ocular toxicities	89% (belantamab mafodotin arm) versus 30% (grade 3/4: 43% (belantamab mafodotin arm) vs. 2%)	[60]
Mirvetuximab soravtansine versus investigator’s choice chemotherapy (FORWARD I)	Phase III, randomized	Platinum-resistant ovarian cancer	Asian % not specified	Blurred vision; keratopathy; dry eye; reduced visual acuity	Blurred vision, 42.0%; keratopathy, 32.5%; dry eyes, 25.9% (mirvetuximab soravtansine arm)	[61]
Mirvetuximab soravtansine versus investigator’s choice chemotherapy (MIRASOL)	Phase III, randomized	FRα-positive platinum-resistant ovarian cancer	Asian 12.3% versus 11.1%	Blurred vision; keratopathy; dry eyes	Blurred vision, 40.8% (grade ≥ 3: 7.8%); keratopathy, 32.1% (grade ≥ 3: 9.2%) (mirvetuximab soravtansine arm)	[62]
Tisotumab vedotin versus investigator’s choice chemotherapy (innovaTV 301)	Phase III, randomized	Recurrent/metastatic cervical cancer	Asian region ~ 34%	Ocular toxicities; conjunctivitis; keratitis; dry eye	Ocular events 52.8% (tisotumab vedotin arm) versus 6.3% (grade ≥ 3: 4.0% (tisotumab vedotin arm) vs. 0)	[63]

*S-1* combination of tegafur, gimeracil, *CAPOX* capecitabine and oxaliplatin, *mFOLFOX6* modified 5-fluorouracil, l-leucovorin, and oxaliplatin, *ATRA* all-trans retinoic acid, *AML* acute myeloid leukemia, *SCC* squamous cell carcinoma, *NSCLC* non-small cell lung cancer, *EGFR* epidermal growth factor receptor

### CTx

Regarding fluoropyrimidine-based regimens, ocular toxicities were inconsistently reported. In a Japanese phase III adjuvant trial for stage III colorectal cancer, S-1 was associated with keratitis (5%; grade ≥ 3, 1%) and epiphora (19%; grade ≥ 3, 2%) [48]. In the same trial, capecitabine was associated with lower incidences of keratitis (2%) and epiphora (5%) [48]. In contrast, most large fluoropyrimidine- or oxaliplatin-based trials did not provide quantitative ocular safety data, although ocular disorders were occasionally listed as adverse events without reporting of incidence [49].

### Hormonal therapy

Hormonal therapy–related ocular toxicities were primarily identified in studies incorporating ophthalmologic follow-up. In a randomized follow-up study of tamoxifen versus toremifene, cataract developed during follow-up in 23.8% of tamoxifen-treated patients [50]. Additionally, a phase III trial of tamoxifen combined with fenretinide reported visual problems in 27% of patients compared with 11.8% in the placebo arm, including grade 3 visual toxicity in 3.5% [51].

### ICIs

Across ICI trials, ocular immune-related adverse events were rarely reported with quantitative incidence. In a phase III trial of nivolumab versus chemotherapy for esophageal squamous cell carcinoma, uveitis was predefined as a criterion for treatment discontinuation; however, its incidence was not reported [52]. Similarly, uveitis was reported as an adverse event of interest with an incidence of less than 1% in the context of pembrolizumab-based perioperative therapy for gastric and gastroesophageal junction cancer [53]. In a Japanese subgroup analysis of adjuvant atezolizumab treatment for resected non–small cell lung cancer, ocular inflammatory toxicity was reported in 1.8% of patients; the percentage was comparable to that associated with best supportive care [54].

### EGFR-targeted therapies

For EGFR tyrosine kinase inhibitors, with the notable exception of dacomitinib, ocular toxicities were infrequently reported. In the ARCHER 1050 trial, conjunctivitis developed in 19% of dacomitinib-treated patients compared with 4% in the gefitinib arm; there were no grade ≥ 3 events in either group [55]. Other EGFR-targeted trials primarily acknowledged ocular considerations only indirectly, such as listing ophthalmologic abnormalities as exclusion criteria [56] or referencing ocular involvement as a baseline metastatic site category [57].

### ADCs

Belantamab mafodotin was associated with the highest frequency of ocular toxicities across multiple phase III trials. In the DREAMM-3 trial, ocular toxicities of special interest occurred in 66% of patients, with grade ≥ 3 events in 29%; blurred vision and keratopathy were reported in 40 and 12%, respectively [58]. Combination regimens further increased the reported incidence: ocular toxicities occurred in 79% of patients in the DREAMM-7 trial (grade ≥ 3, 34%) [59] and in 89% in the DREAMM-8 trial (grade 3/4, 43%) [60]. Mirvetuximab soravtansine treatment for ovarian cancer was also frequently associated with ocular toxicities. In the FORWARD I trial, blurred vision, keratopathy, dry eye, and reduced visual acuity were reported in 42.0, 32.5, 25.9, and 19.3% of patients, respectively [61]. These findings were confirmed in the MIRASOL trial, where blurred vision (grade ≥ 3, 7.8%), keratopathy (grade ≥ 3, 9.2%), and dry eye were reported in 40.8, 32.1, and 28.0% of patients, respectively [62]. Similarly, tisotumab vedotin used in the management of recurrent or metastatic cervical cancer was associated with a high incidence of ocular toxicities. In the innovaTV 301 trial, ocular toxicities occurred in 52.8% of patients compared with 6.3% in the investigator’s choice chemotherapy arm, with grade ≥ 3 ocular toxicities reported in 4.0% of patients [63].

Ocular/visual toxicities were extracted only from trials in which such events were explicitly reported or categorized as adverse events of special interest. When incidence was not quantitatively reported, this is explicitly stated to highlight variability in adverse event reporting across trials.

### Proposed mechanisms of anticancer drug–associated ocular toxicities

Anticancer drug–associated ocular toxicities arise through several distinct but partially overlapping mechanistic pathways that reflect drug-specific pharmacology and tissue-specific vulnerabilities within the eye. Broadly, these mechanisms include direct cytotoxic injury to rapidly proliferating ocular surface epithelium, as observed with fluoropyrimidines and cytarabine [34, 68], as well as payload-driven off-target epithelial toxicity associated with certain ADCs [69]. Platinum agents are more commonly linked to vascular and neuroretinal ischemic injury, including optic neuropathy and retinal circulation impairment [70, 71]. In contrast, ICIs induce T-cell–mediated inflammatory processes resulting from immune dysregulation and loss of ocular immune privilege [72]. Targeted therapies, including EGFR/FGFR and MEK inhibitors, disrupt epithelial maintenance and retinal fluid homeostasis, leading to reversible barrier dysfunction and serous retinal changes [73, 74]. Together, this mechanistic framework explains differences in anatomical predilection, reversibility, and clinical management across therapeutic classes.

### Recommendations for the management of anticancer drug–associated ocular toxicities: Asian ethnicity–specific considerations

Due to differences in treatment patterns, drug availability, and potentially ocular anatomical characteristics, management recommendations for Asian patients should be derived from clinical trials that specifically include or evaluate Asian populations. Furthermore, considering the higher HLA-DR4 prevalence in Asian populations and increasing evidence linking HLA-DR4 to Vogt–Koyanagi–Harada–like uveitis, clinicians should be vigilant for the occurrence of more severe uveitis inflammatory forms in these patients [10–12]. Clinicians in Asian settings should maintain a high index of suspicion, particularly for fluoropyrimidine- and platinum-based regimens, and adopt proactive symptom-based monitoring strategies.

It must be emphasized that the symptom-based management proposed in this review was developed through a synthesis of available clinical data, case reports, and expert consensus, rather than a systematic evaluation of high-level evidence from randomized controlled trials, which is currently lacking in this field. Therefore, these algorithms have not been prospectively validated and should be regarded as preliminary guidance. They are intended to provide a practical framework for non-ophthalmologists to facilitate early detection and standardize specialist referral; they should not be viewed as definitive evidence-based mandates. Clinicians should always exercise clinical judgment and adapt these suggestions to the specific oncological setting and individual patient needs. These recommendations are generally aligned with the recommendations in the current guidelines [75, 76].

It should be noted that robust prospective evidence regarding the management of these ocular toxicities in Asian patients is currently limited. Therefore, these proposed management algorithms and recommendations are largely based on the authors’ clinical experience and systematic interpretation of the existing literature, representing a practical expert consensus for non-ophthalmologic settings.

### General principles applicable to non-ophthalmologic settings

Anticancer drug–associated ocular toxicities encompass a broad spectrum of clinical manifestations, ranging from mild ocular surface symptoms to visual impairment. As demonstrated in the present literature review, the availability of high-level evidence supporting standardized management strategies remains limited, particularly for CTx and MTAs. Moreover, many published recommendations rely on ophthalmologic examinations or specialized diagnostic equipment, which may not be readily available in non-ophthalmologic settings. In Asian countries, including Japan, anticancer regimens such as fluoropyrimidines (e.g., S-1 and capecitabine) and platinum-based chemotherapy are frequently used [77, 78], wherein several ocular toxicities have been reported predominantly from Asian cohorts. These regional treatment patterns underscore the need for pragmatic, symptom-oriented management approaches that can be implemented even in facilities without on-site ophthalmologists.

### Baseline assessment and patient education

Before initiating anticancer therapy, clinicians should obtain a focused ocular history, including pre-existing eye disease, prior ocular surgery, contact lens use, and baseline visual symptoms. Formal ophthalmologic screening is not mandatory for all patients; however, baseline documentation of visual complaints may facilitate early detection of treatment-emergent symptoms. Patients should be informed that ocular symptoms, such as blurred vision, visual field disturbances, eye pain, redness, tearing, or photophobia, may occur during treatment. In Asian populations, where mild symptoms may be underreported, explicit encouragement to report even transient or intermittent visual disturbances is particularly important.

### Symptom-based monitoring during treatment

Routine monitoring should primarily rely on patient-reported symptoms and basic physical inspection, rather than specialized testing. In each treatment cycle, clinicians and medical staff should actively inquire about new or worsening ocular symptoms. Simple bedside assessments, such as gross visual acuity comparison between eyes or inspection for conjunctival injection and excessive tearing, may be performed when feasible but are not mandatory. This symptom-based strategy is particularly relevant in non-ophthalmologic settings, where access to slit-lamp examination, visual field testing, or optical coherence tomography is limited.

### Initial management in non-ophthalmologic settings

For patients presenting with mild ocular symptoms (e.g., eye pain, light sensitivity, eye discomfort, dryness, tearing, or transient blurred vision), conservative management is recommended as the first step.

- S-1–related experimental and translational studies have suggested an association between tear drug concentration and ocular toxicity, supporting the rationale for tear dilution strategies. Therefore, artificial tears (applied approximately 4 times a day) may be initiated empirically, particularly for fluoropyrimidine-associated ocular surface symptoms.
- Temporary observation without treatment modification is acceptable if symptoms are mild, transient, and non-progressive.

For moderate symptoms (e.g., recurrent visual disturbances, persistent blurred vision, or functional impairment), escalation of supportive care should be considered, and early referral to ophthalmology is recommended.

For institutions without an on-site ophthalmologist, the administration of high-risk anticancer agents should be contingent upon the following safety requirements: (1) establishment of a formal collaboration system with a rapid referral pathway to a nearby specialized ophthalmology clinic; (2) mandatory pre-administration screening to assess baseline ocular health and identify pre-existing conditions (e.g., dry eye, corneal disorders) that may increase the risk of toxicity; (3) implementation of clear, symptom-based referral criteria as outlined in our proposed algorithms; and (4) construction of a multidisciplinary team-based support system involving oncologists, pharmacists, and nurses to ensure continuous monitoring and patient education. For specific high-risk agents requiring specialized monitoring—such as belantamab mafodotin and mirvetuximab soravtansine (which require frequent slit-lamp exams for keratopathy) or MEK inhibitors (which require retinal evaluation)—direct co-management with an ophthalmologist is mandatory. In settings where an on-site ophthalmologist is unavailable, these agents should only be administered if a stringent, pre-arranged collaborative system is in place to ensure that all regulatory-mandated ophthalmological assessments are strictly performed. If such a specialized referral and monitoring system cannot be guaranteed, the administration of these specific high-risk medications should be avoided in that setting.

### Drug class–specific considerations

#### Ocular toxicities induced by CTx

#### S-1–associated ocular toxicities

S-1 is frequently associated with lacrimal drainage obstruction and corneal epithelial damage, particularly in Asian populations. An experimental canine study demonstrated that repeated administration of artificial tears reduced S-1–induced corneal surface injury, suggesting a potential preventive role for lubrication therapy [22]. Furthermore, a clinical pharmacokinetic study showed that higher tegafur concentrations in tear fluid were associated with ocular symptoms, including lacrimal obstruction and corneal disorders [21]. Although this study did not evaluate therapeutic interventions, it supports a drug exposure–dependent mechanism.

#### Recommended management (non-ophthalmologic settings)

- Prophylactic or early initiation of artificial tears (applied approximately 4 times a day) in symptomatic patients
- Education regarding early symptoms (e.g., tearing and foreign body sensation)
- Dose interruption or modification if corneal symptoms progress
- Ophthalmologist consultation when visual acuity declines or symptoms persist despite lubrication

#### Capecitabine-associated ocular toxicities

Capecitabine has been associated with various ocular toxicities, including visual impairment [23], bilateral ectropion [24], conjunctivitis [25], and corneal toxicity [26, 27]. No reports examining prevention or treatment were found. Empirical management is recommended.

#### Recommended management

- Symptom-based monitoring and visual acuity testing
- Artificial tears (applied approximately 4 times a day) for mild conjunctival or corneal symptoms
- Drug interruption and referral for progressive corneal involvement or eyelid malposition

#### Oxaliplatin-associated ocular toxicities

Oxaliplatin-induced visual disturbances, including transient or irreversible visual loss, have been reported in multiple case reports [29–33] and one retrospective study [28]. No reports examining prevention or treatment were found. Empirical management is recommended.

#### Recommended management

- Immediate assessment of visual acuity upon symptom onset
- Temporary discontinuation of oxaliplatin in case of acute visual loss
- Avoidance of rechallenge in cases of severe or recurrent symptoms

#### Cytarabine-associated ocular toxicities

Cytarabine-induced ocular toxicities, particularly conjunctivitis and keratitis, are well recognized [34–37]. Prophylactic and therapeutic use of topical corticosteroids has been reported to reduce symptom severity [34–37].

#### Recommended management

- Prophylactic or early therapeutic steroid eye drops when cytarabine is administered at high doses
- Artificial tears (applied approximately 4 times a day) as adjunctive therapy
- Ophthalmologist consultation for persistent or severe keratitis

### MTA and ADCs

#### Management considerations

Currently, no prospective or interventional studies have evaluated preventive or therapeutic strategies for MTA- or ADC-induced ocular toxicities. Literature consists exclusively of expert consensus and management recommendations [43–47]. Notably, an exception exists for tisotumab vedotin, for which prospective clinical trials incorporated prophylactic ocular interventions, including lubricating eye drops, steroid eye drops, vasoconstrictor drops, and cooling eye masks, to mitigate corneal and conjunctival toxicities [63]. Overall, evidence-based preventive or therapeutic strategies for ocular toxicities related to MTA and ADCs remain limited, and current management approaches continue to rely primarily on early symptom recognition, empirical supportive care, and prompt ophthalmologic referral when indicated.

#### Recommended management

- In principle, it should be administered under the supervision of an ophthalmologist.
- Early recognition of dry eye, blurred vision, or photophobia
- Empirical initiation of artificial tears
- Treatment interruption for persistent or moderate symptoms
- Ophthalmologist consultation when symptoms do not improve or visual acuity declines

#### ICI–associated ocular toxicities

For ICI–associated ocular inflammatory disorders, topical or systemic corticosteroids have been reported to be effective [41, 42].

#### Recommended management

- Prompt recognition of inflammatory symptoms (redness, pain, and photophobia)
- Steroid eye drop use for suspected immune-related ocular inflammation
- Multidisciplinary discussion regarding immunotherapy continuation
- Ophthalmologist consultation when feasible

#### Simplified algorithm for facilities without ophthalmologists

- Identify drug class
- Assess symptoms and visual acuity
- Initiate artificial tears for mild symptoms
- Use steroid eye drops for suspected inflammatory conditions
- Interrupt anticancer therapy for acute or progressive visual impairment
- Consult an ophthalmologist when symptoms persist or worsen

#### Recommendation of topical steroid therapy

- Topical (First-line): For mild to moderate inflammatory AE (e.g., ICI-induced uveitis), 0.1% fluorometholone or 0.1% betamethasone eye drops administered 4 times daily are typically initiated.
- Systemic (Severe): For Grade 3–4 immune-related ocular toxicities (e.g., optic neuritis or severe uveitis), systemic prednisolone (0.5 to 1.0 mg/kg/day) should be considered in close consultation with an ophthalmologist.

### Thresholds

Referral to Ophthalmology: Prompt referral is recommended for any symptoms corresponding to CTCAE Grade 2 or higher (e.g., symptomatic vision loss, moderate pain, or interference with activities of daily living) or any persistent unexplained ocular redness or discomfort.

Treatment Interruption: Consider temporary discontinuation for Grade 3 toxicities. For certain agents like ADCs (e.g., T-DXd or belantamab mafodotin), treatment should be held until toxicity resolves to Grade 1 or baseline, as per the manufacturer’s prescribing information.

For endocrine therapies, evidence supporting specific preventive or management strategies for ocular toxicities is lacking. Therefore, no drug class–specific recommendations are proposed in this review, and clinical management should be individualized based on symptom severity and ophthalmologic assessment.

## Discussion

### Asian ethnicity–specific considerations in anticancer therapy–associated ocular toxicities

Ethnic differences in ocular anatomy and physiology may influence the incidence and clinical manifestations of anticancer therapy–associated ocular toxicities. Compared with other populations, Asian individuals show distinct ocular surface characteristics, including differences in eyelid anatomy, tear film stability, and corneal structure, which may predispose them to specific ocular toxicities or alter symptom perception and severity. Despite these known anatomical and physiological differences, most existing studies do not stratify ocular toxicity data by ethnicity, making it difficult to directly compare Asian and non-Asian populations. However, pharmacokinetic and observational data suggest that drug exposure at the ocular surface may play a role in toxicity development. For example, in S-1–treated patients, higher tegafur concentrations in tear fluid have been associated with lacrimal drainage obstruction and corneal disorders, supporting a mechanism of local drug exposure–related toxicity [21]. Experimental data from canine models further suggest that repeated artificial tear administration may reduce S-1–induced corneal epithelial damage, indicating a potentially practical preventive strategy applicable to Asian clinical settings [22]. Given that many Asian oncology centers manage high patient volumes with limited ophthalmologic resources, the development of management strategies tailored to Asian healthcare systems and patient characteristics is particularly important.

### Implications for management in non-ophthalmologic oncology settings

An important finding of this review is the gap between published management recommendations and real-world feasibility in institutions without on-site ophthalmologists. While preventive and therapeutic strategies for cytarabine-induced ocular toxicity, such as prophylactic or early steroid eye drops, have been supported by clinical studies and incorporated into routine practice [34–37], similar evidence-based approaches are lacking for most other CTx. For ICI–associated ocular toxicities, limited case reports suggest that topical steroid therapy can be effective against inflammatory manifestations such as conjunctivitis or uveitis [41, 42]. However, standardized protocols suitable for non-specialist settings remain undeveloped. These findings underscore the need for simplified, symptom-based algorithms that rely on minimal diagnostic requirements, such as patient-reported symptoms and basic visual acuity testing, which prioritize early intervention and timely referral when necessary.

### Future research directions

Future research should prioritize prospective observational studies and interventional trials specifically designed to evaluate preventive and management strategies for anticancer therapy–associated ocular toxicities. Such studies should include:

- Ethnicity-stratified analyses, particularly focusing on Asian populations, to clarify differences in incidence, severity, and treatment response.
- Pragmatic intervention studies assessing low-cost, widely available measures, such as artificial tears or prophylactic topical therapies, in both cytotoxic and targeted therapy–related ocular toxicities.
- Implementation-focused research evaluating management algorithms suitable for oncology settings without specialized ophthalmologic support.

Addressing these gaps would facilitate the development of evidence-based, context-appropriate management strategies, ultimately improving patient QOL while ensuring the safe continuation of anticancer therapy.

Limitations.

This review has limitations that should be acknowledged. First, most of the included studies consist of lower-level evidence, such as case reports, small case series, and retrospective analyses, rather than prospective randomized controlled trials. This is particularly evident for certain cytotoxic agents such as oxaliplatin and capecitabine [23–33]. Consequently, the synthesized data are inherently susceptible to selection and reporting biases; for instance, severe or unusual ocular complications may be overrepresented in the literature, while milder, self-limiting toxicities might be underreported.

Second, there is a notable lack of studies specifically designed to evaluate preventive or therapeutic interventions for ocular toxicities, particularly for molecular targeted agents and ADCs. Current management recommendations often rely on clinical experience and expert consensus [43–47], or extrapolation from general ophthalmologic practice, such as the empirical use of artificial tears. To address this, our proposed management algorithms are intended as expert-based practical frameworks rather than definitive evidence-based mandates.

Third, the applicability of these recommendations may be influenced by clinical settings. Many guidelines presuppose access to specialized ophthalmologic equipment (e.g., slit-lamp evaluation), which may not be readily available in all oncology departments. Our review attempts to bridge this gap by focusing on early detection and timely referral.

Fourth, although we focused on Asian populations, the available evidence is characterized by significant heterogeneity. The term “Asian population” in this review encompasses diverse ethnicities (East, South, and Southeast Asian), and data were often derived from specific geographic regions or subgroup analyses of global trials. Due to the lack of head-to-head comparative data and the high heterogeneity of study designs, a formal stratified analysis or risk-of-bias assessment for each study was not feasible. Furthermore, while pharmacological factors like CYP2A6 polymorphisms support ethnicity-specific differences, other variations may stem from regional differences in clinical practice or healthcare access.

Finally, our literature search was limited to the PubMed database and English-language publications, which may introduce language and selection bias. Relevant studies published in local Asian languages or indexed in other databases, such as Embase or the Cochrane Library, may have been excluded. Although we supplemented our search with a manual reference review, future studies incorporating multilingual sources and broader databases are warranted to provide a more comprehensive global perspective.

## Data Availability

The data supporting the review findings are available from the corresponding author, YI, upon reasonable request.
